# The Editor's Letter-Box

**Published:** 1919-01-11

**Authors:** H. McKinley

**Affiliations:** 6 Westborough Road, Westcliff.


MY LORD,—In a letter which appeared in THE HOSPITAL of August 3 last I stated that the London Hospital sends out to private cases nurses who have been in training at the hospital for less than one year.

It was in the full belief that this charge was true and that I was performing a public duty that I made this statement. Events have, however, proved it to be without foundation, and I desire to make a complete and unqualified withdrawal and to express my regret that I made the statement referred to.—Yours, &c.,

H. MCKINLEY.

6, Westborough Road, Westcliff.

Comment is needless.—K.

